# HIV taken by STORM: Super-resolution fluorescence microscopy of a viral infection

**DOI:** 10.1186/1743-422X-9-84

**Published:** 2012-05-02

**Authors:** Cândida F Pereira, Jérémie Rossy, Dylan M Owen, Johnson Mak, Katharina Gaus

**Affiliations:** 1 Centre for Vascular Research, University of New South Wales, Sydney, Australia; 2 Centre for Virology, Burnet Institute, Melbourne, Australia; 3 Monash Micro Imaging, Clayton, Australia; 4 Department of Medicine, Monash University, Clayton, Australia; 5 School of Medicine, Deakin University, Geelong, Australia; 6 Commonwealth Scientific and Industrial Research Organization, Australian Animal Health Laboratory, Geelong, Australia

**Keywords:** HIV, Super-resolution microscopy, Electron microscopy, Viruses, Dynamic movement, Protein rearrangement

## Abstract

**Background:**

The visualization of viral proteins has been hindered by the resolution limit of conventional fluorescent microscopes, as the dimension of any single fluorescent signal is often greater than most virion particles. Super-resolution microscopy has the potential to unveil the distribution of proteins at the resolution approaching electron microscopy without relying on morphological features of existing characteristics of the biological specimen that are needed in EM.

**Results:**

Using direct stochastic optical reconstruction microscopy (dSTORM) to achieve a lateral resolution of 15–20 nm, we quantified the 2-D molecular distribution of the major structural proteins of the infectious human immunodeficiency virus type 1 (HIV-1) before and after infection of lymphoid cells. We determined that the HIV-1 matrix and capsid proteins undergo restructuring soon after HIV-1 infection.

**Conclusions:**

This study provides the proof-of-concept for the use of dSTORM to visualize the changes in the molecular distribution of viral proteins during an infection.

## Background

The human immunodeficiency virus type 1 (HIV-1) is approximately spherical with a mean diameter of 125 ± 14 nm [1-3]. Its main structural components are a lipid bilayer containing envelope glycoproteins, a matrix shell located beneath the viral lipid membrane and a capsid core with a cone shaped geometry [1-3]. After fusion of HIV-1 with the target cell, it is postulated that the matrix shell is retained at the plasma membrane while the capsid core undergoes a dramatic disassembly process that facilitates the reverse transcription of the viral genome [3-5]. Fluorescent labeling of HIV-1 proteins has provided valuable insights into their sub-cellular localization in infected cells [6,7]. However, the molecular mechanisms of cell entry and replication are difficult to detect since the viral particle is smaller than the resolution limit of conventional fluorescent microscopes. Electron microscopy techniques have provided structural insights of HIV-1 [1,2,7] but remain technically demanding and are prone to introduce artifacts.

The development of far-field super-resolution light microscopy methods such as stochastic optical reconstruction microscopy (STORM) [8] and photoactivatable localization microscopy (PALM) [9] enable the localization of individual photoswitchable protein-labeled molecules with a spatial resolution of tens of nanometers. The more recently developed direct STORM (dSTORM) combines standard immunocytochemistry, total internal reflection fluorescence (TIRF) microscopy and reversible photoswitching of conventional organic fluorochromes such as Cy5 and Alexa 647 to further improve the quality of signals [10]. This technique has a tremendous potential for the visualization of the molecular organization of viral proteins during an infection, particularly if tagging with fluorescent proteins compromises virus infectivity. Previously studies have used super-resolution techniques to visualize clusters of viral proteins artificially transfected into cell lines [11-13]. In this study we have used dSTORM to provide one of the first the molecular distribution of matrix and capsid proteins in infectious HIV-1 particles before and after a real infection of lymphoid cells.

## Results and discussion

**Figure 1:**
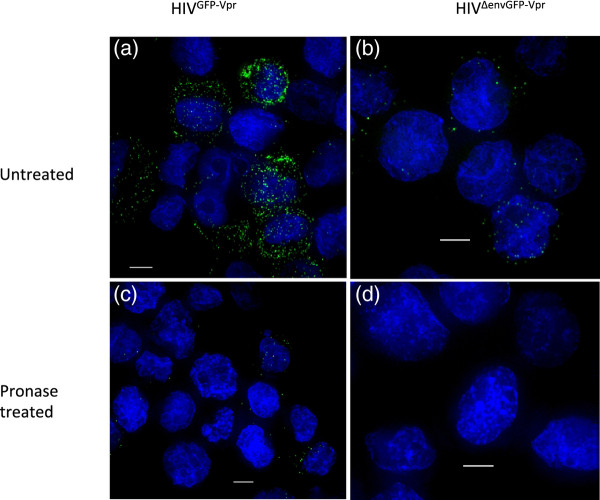
**Pronase treatment removes non-internalized virus particles from the cell surface.** MT-2 cells were infected with (**a, c**) HIV^GFP-Vpr^or (**b, d**) HIV^ΔenvGFP-Vpr^ for 2 h at 17°C to allow binding of the virions to the cells. Afterwards the cells were washed to remove unbound virus particles and incubated for 20 min at 37°C to allow virus entry into the cells. The samples were then split and half of the cells were incubated with PBS (**a-b**) while the other half was treated with pronase (**c-d**) to remove non-internalized virus particles. Subsequently, all samples were fixed, counterstained, mounted and visualized by widefield microscopy followed by deconvolution. GFP is shown in green and nuclei in blue. The provided images were derived from a volume compression of a z stack of 28 images taken at a 0.3-μm step size. Scale bar, 5 μm. Images are representative of 3 independent experiments.

We used dSTORM to visualize matrix and capsid proteins in cell-free HIV-1 virions and in infected lymphoid cells. Similar to previously describe [7,14], we first generated HIV-1 particles that contained the green fluorescent protein-viral protein R fusion protein (HIVGFP-Vpr), which allows us to visualize particles with conventional fluorescence microscopy. The same virion preparation was then either evenly spread onto glass coverslips or used to infect lymphocytes. Importantly, non-internalized viral particles were removed from the surface of the target cells by pronase treatment twenty minutes after the virus was allowed to enter the target cells. This was confirmed by incubation of the target cells with an envelope-deficient HIV-1, which was unable to enter the target cells and therefore was cleaved by the pronase and consequently no viral protein could be detected in these samples (Figure 1). Therefore, all matrix and capsid protein clusters associated with the lymphocytes were internalized. Infected cells were fixed and plated onto glass coverslips by cytospin centrifugation. Both samples were immuno-stained with antibodies recognizing either the matrix or caspid protein.

**Figure 2:**
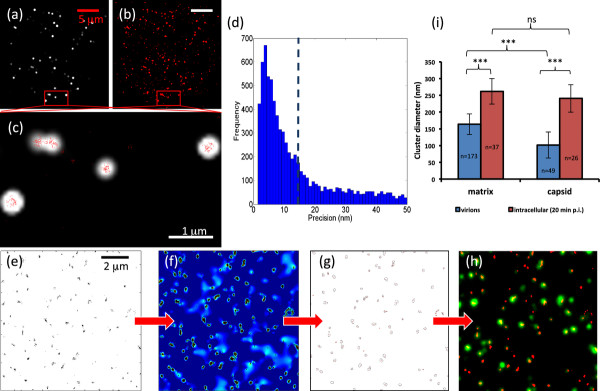
**Super-resolution imaging of individual molecules of infectious HIV-1 before and after entry into lymphocytes.** (**a-c**) Conventional total internal reflection fluorescence (TIRF) image (**a**), corresponding dSTORM image (**b**) and overlay of the TIRF (white) and dSTORM (red) images (**c**) of the HIV-1 matrix protein 20 minutes after synchronized entry into the lymphoid cell line MT-2. (**d**) Histogram of the localization precision values of molecular coordinates localized by dSTORM corresponding to the data set shown in **a-c**. Localization precision corresponds to one sigma of the Gaussian distribution of the point spread function that is fitted to individual molecules and is also affected by photons and noise level. Dashed line indicates mean. (**e-h**) Cluster analysis of the matrix protein in cell-free virions based on Ripley’s *K*-function converts the point distribution of molecular coordinates (**e**) into a cluster map with highly to less clustered regions colored red to blue (**f**). Cluster statistics such as number, size and associated molecules were extracted from thresholded images (**g**). By overlaying the TIRF image of GFP-Vpr (green) with the binary cluster map, the association of viral proteins with the reverse transcription complex after cell entry was quantified (**h**). Scale bars, 5 μm in panels in **a-b**; 1 μm in panel **c** and 2 μm in panels **e-h**. (**i**) Quantitative analysis of the diameter of the molecular clusters of capsid proteins in cell-free virions and 20 min post infection into MT-2 cells. Error bars represent the standard deviation of the mean from 26–173 clusters per sample from a representative from two experiments. *** = *p* < 0.001; ns = non-significant.

We first compared conventional to super-resolution images. In TIRF images, HIV-1 proteins in infected T-lymphocytes appeared as bright punctuate structures (Figure 2a). In dSTORM, the stochastic activation of fluorophores allows the analysis of the point-spread function (PSF) of individual proteins. In addition to the x-y localization, the fitting algorithms also return the localization precision, number of photons emitted per molecules, and background values associated with each molecule. These characteristics allow us to apply stringent conditions for single molecule detection [15] and standardize the image quality across the experimental conditions. When the molecular coordinates of the individual matrix proteins are plotted in an image, it becomes apparent that dSTORM revealed a greater heterogeneity in distribution of the same protein than in TIRF images (Figure 2b). The increase in resolution achieved with dSTORM is illustrated by the overlay of the two images with protein clusters in dSTORM appearing significantly smaller than the conventional image (Figure 2c). The molecular localization precision of the dSTORM approach was 15–20 nm (Figure 2d).

To quantify the distribution and heterogeneity of viral proteins, we used a variation on Ripley’s K- function analysis [16]. As shown for the matrix protein in cell-free HIV-1 (Figure 2e-h), the single molecule dSTORM image was converted into a pseudo-colored cluster map that is based on the number of other molecules within a 50 nm radius, normalized to the overall particle density (Figure 2f). The contours describing each cluster were extracted and the number of clusters and cluster sizes determined (Figure 2g). To identify the replicating viruses that are travelling to the nucleus we used the GFP-Vpr fusion protein, which is incorporated into the virus particles and remains associated with the replicating viruses during intracellular trafficking [7]. GFP-Vpr was therefore used to identify ‘double positive’ viral protein clusters that were associated with the viral replication machinery and contained the matrix or capsid protein (Figure 2h).

This analysis allowed us to accurately measure the sizes of matrix and capsid protein clusters co-localized with GFP-Vpr in cell-free virons and in infected cells (Figure 2i). In cell-free virions, the size of the matrix protein clusters are within the expected range of 106–183 nm [1] and, as expected, the size of the capsid protein clusters are significantly smaller than the matrix protein clusters (*p* < 0.0001, Figure 2i). Twenty minutes post infection of T lymphocytes, the matrix protein clusters and the capsid protein clusters were similar in size (Figure 2i), which indicates that upon infection the capsid protein clusters showed a significantly large fractional increase in size (236%) when compared with the matrix protein clusters. This single molecule imaging approach hence allowed us to follow the restructuring of the matrix shell and capsid core during infection, which may reflect the structural rearrangements that facilitate the HIV-1 reverse transcription process, such as virion uncoating.

## Conclusion

We were able to quantify the size of the HIV-1 matrix shell and capsid core by dSTORM and these results were in agreement with the known HIV organization seen by EM [1,2,17]. Furthermore, this approach provided new information showing that upon cell entry, the size of the virion matrix shell and capsid core increased significantly when compared to cell-free HIV-1 virions, which indicates that the HIV particles underwent a dramatic rearrangement immediately after entry into the target cell. In summary, this study validates the use of dSTORM to assess the molecular distribution of viral proteins during the life-cycle of an infectious virus, and it opens up new possibilities to study the distribution and re-distribution of viral proteins at the early phase of viral infection.

## Methods

### Cells and virus

MT-2 cells (obtained through the AIDS Research and Reference Reagent Program, Division of AIDS, NIAID, NIH from D. Richman) [18,19] were cultured in Rosewell Park Memorial Institute (RPMI) 1640 medium (Invitrogen) supplemented with 10% vol/vol heat-inactivated fetal calf serum (FCS; Invitrogen, Mount Waverley, Victoria, Australia) and penicillin/streptomycin. 293 T cells were maintained in Dulbecco’s modified Eagle medium/high modified (with 4500 mg/l dextrose and 4 mM L-glutamine) medium (DMEM; Invitrogen), supplemented with 10% (vol/vol) heat-inactivated cosmic calf serum (CCS; Hyclone, Tauranga, New Zealand), 100 U/ml of penicillin and 100 mg/ml of streptomycin (Invitrogen).

The pNL4-3 proviral DNA (obtained through the AIDS Research and Reference Reagent Program, Division of AIDS, NIAID, NIH from M. Martin [20]) contains the NL4-3 infectious molecular clone of HIV-1. The pNL4-3^Δenv^ proviral DNA (obtained through the AIDS Research and Reference Reagent Program, Division of AIDS, NIAID, NIH from N. Landau [21,22]) contains an envelope defective-pNL4-3 molecular clone of HIV-1. HIV-1 particles were produced by poly(ethylenimine) (PEI; Polysciences Inc., Warrington, PA, USA) transfection of 293 T cells with pNL4-3 or pNL4-3^Δenv^ proviral DNA and GFP-Vpr plasmid (kindly provided by T. Hope, Northwestern University) to generate GFP-Vpr-labeled HIV-1 (HIV^GFP-Vpr^or HIV^ΔenvGFP-Vpr^). Forty hours post-transfection viral particles were purified, concentrated and quantified as previously described [14]. Briefly, supernatant from 293 T cells was filtered and viral particles were concentrated by ultracentrifugation through a 20% sucrose cushion at 100,000 x g for 1 h at 4°C using an L-90 ultracentrifuge (SW 41 rotor; Beckman, Fullerton, CA, USA) and virus pellets were resuspended in 1x phosphate buffered saline (PBS; Invitrogen) and quantified using a HIV-1 antigen (p24 CA) micro enzyme-linked immunosorbent assay (ELISA) (Vironostika: Biomerieux, Boxtel, The Netherlands).

### Infection of lymphoid cells

Synchronized infections were performed as described previously [23]. Briefly, MT-2 cells were infected with HIV^GFP-Vpr^ (normalized to 1000 ng of p24 per million cells) by spinoculation at 17°C for 2 h at 1,200 x g. Afterwards, the cells were washed twice with PBS to remove unbound virus and incubated with warm media at 37°C, 5% CO2 for 20 min to initiate infection. Afterwards, the cells were washed, treated with 2 mg/ml of protease from *Streptomyces griseus* (pronase E; Sigma-Aldrich, Castle Hill, NSW, Australia) for 10 min on ice and washed extensively with PBS containing 20% FCS. The cells were then fixed with 4% formaldehyde (Polysciences) in 0.1 M pipes buffer, pH 6.8, washed with PBS and cytospined onto glass coverslips. Cell-free viruses (same batch as used for the infection of lymphoid cells) were also fixed with formaldehyde in pipes buffer, evenly spread on glass slides to achieve optimal sample thickness, incubated at 4°C for 16 h and washed twice with PBS.

### Immunofluorescence staining

Cells and virus were permeabilized and stained with mouse anti-matrix (SVM-33) antibody (MH-SVM33C9, ATCC, Manassas, VA (Akzo Nobel N.V.) or mouse anti-capsid (AG3.0) antibody (obtained through the AIDS Research and Reference Reagent Program, Division of AIDS, NIAID, NIH from J. Allan) [24] and goat Cy5-conjugated anti-mouse secondary antibody (Jackson ImmunoResearch, USA).

### Image acquisition and analysis

Antibody stained cells were imaged in an oxygen scavenging buffer (50 μ g/ml glucose oxidase, 25 μ g/ml horseradish peroxidase, 75 mM β-mercaptoethylamine, 25 mM Hepes, 25 mM glucose, 5% glycerol in PBS, pH 8) in an open Chamlide™ chamber (Live Cell Instrument, Seoul, Korea). Cells were imaged with surface-immobilized 100 nm colloidal gold beads (BBInternational, Cardiff, UK) that allow correction for sample drift during the acquisition.

dSTORM images were acquired on a prototype PALM microscope (Carl Zeiss GmbH, Jena, Germany) with TIRF illumination. In dSTORM, the carbocyanine dye Cy5 is stochastically converted to a long-lived dark state (‘off’) when excited using 633 nm (15 mW) laser radiation and switched back ‘on’ by exposure to low intensities of 488 nm (0.1–1 mW) laser light when the sample is immersed in a oxygen depleted buffer containing a reducing agent [10]. By adjusting the intensity of the 488 nm laser, the density of fluoroescent molecules was approximately kept constant during acquisition and across samples. Images of 5–6 cells per sample from two different experiments were captured using an Andor iXon DU-897D EMCCD camera (Andor Technology Plc, Belfast, UK), giving a pixel size of 100 nm at the sample plane.

dSTORM images were reconstructed from a series of 20,000 TIRF images using Zeiss Zen software. Molecular clustering was analyzed using Getis and Franklins 2^nd^ order analysis as previously described [25]. Localization precision corresponds to one sigma of the Gaussian distribution of the point spread function that is fitted to individual molecules and is also affected by photons and noise level [16]. Data was cropped so as to exclude points with localization precision worse than 50 nm. A 10 × 10 μm region is then selected for analysis and rendered into cluster maps with 7 nm/pixel resolution. Cluster maps were threshold to create a binary map from which only clusters that significantly overlapped with a TIRF image of GFP-Vpr were selected and analyzed using ImageJ [26].

### Pronase treatment efficiency

MT-2 cells were infected with HIV^GFP-Vpr^ or HIV^ΔenvGFP-Vpr^ as described above and afterwards the samples were split. Half of the cells were treated with pronase to remove non-internalized virus as described above and half of the cells were incubated with PBS. The cells were then cytospined onto glass slides, counterstained with Hoechst 33258 (Invitrogen), mounted in Fluoromount-G (Electron Microscopy Sciences, Hatfield, PA) and images were captured in a z series on a charge-coupled device (CCD) camera (CoolSnap HQ; Photometrics, Tucson, AZ) through a 100 × 1.4 numerical aperture (NA) oil immersion lens on a DeltaVision microscope (Applied Precision, Issaquah, WA) and deconvolved using softWoRx deconvolution software (Applied Precision).

### Statistical analysis

Data derived from the diameter of 26–173 molecular clusters per sample was analyzed by paired two-tailed Student’s *t* test. A *p* value < 0.001 was considered highly statistically significant for all tests.

## Competing interests

The authors declare that they have no competing interests.

## Author’s contributions

CFP carried out the virus components of this study in the Mak lab (JM) at Burnet Institute; JR and DMO conducted the dSTORM imaging and analyses in the Gaus lab (KG) at UNSW. All authors contributed to the design, data interpretation and writing of the manuscript. All authors read and approved the final manuscript.
